# Supplementary material for: Shifts in Diabetes Health Literacy Policy and Practice in Australia—Promoting Organisational Health Literacy
Source: Int J Environ Res Public Health. 2023 May 10;20(10):5778. doi: 10.3390/ijerph20105778 (PMC10217931; doi:10.3390/ijerph20105778)
Supplement: Supplementary file 1 [file ijerph-20-05778-s001.zip › ijerph-2253581-supplementary.pdf]

Table S1: Research design and timeline.

								Mar-22	Apr-22				
		Sep-21	Oct-21	Nov-21	Dec-21	Jan-22	Feb-22	SL,RK	SL,RK,DM	May-22	Jun-22		
		GC, GM	GC, GM	GC, GM	GC, GM	GC, GM	GC, GM		Apr-22	GM, SL, DM	GM, SL, DM	Jul-22	Aug-22
Jul-21	Aug-21	Sep-21	Oct-21	Nov-21		Jan-22	Feb-22	Mar-22	GC, GM	May-22	Jun-22	GM, SL, DM	SD, SL, GM, GC, JR, DM
GM	GM, SL, DM	SD, SL, GM, GC, JR, DM	GC, GM	GC, GM, SD		GC, GM	SD, SL, GM, GC, JR, DM	SD, SL, GM, GC, JR, DM		SD, SL, GM, GC, JR, DM	SD, SL, GM, GC, JR, DM		
↑	↑	↑	↑	↑	↑	↑	↑	↑	↑	↑	↑	↑	↑
Study was conceptual-ised and presented to potential research team and key stakeholders	Blue print of research process and publication strategy determined and finalised by research team.	1) Initial literature search on Medline and CINHAL using search terms 'diabetes' and 'organisation al health literacy' (no peer-reviewed literature identified).<br><br>2) Search of the NDSS, Diabetes Australia and Australian Government websites for reports, position statements or policies on diabetes and health literacy.	1) Time frame determined – (2006 to 2021).<br><br>2) A search was performed for the patient fact sheets available for all relevant time periods: 2006, 2010, 2016 and 2021.<br><br>3) Random excel generation of selected fact sheets and fact sheets randomly selected for PEMAT analysis.	1) Listing of policies, position papers and reports identified on excel.<br><br>2) Summary describing the health literacy challenge and the organisation al response taken or practice change made to improve health literacy was listed.	1) Preliminary themes identified for diabetes health literacy challenge.<br><br>2) A narrative was prepared for each theme to explain the historical activities taking place.<br><br>3) A draft definition was allocated to each theme.<br><br>4) Relevant OHL responses identified that correspond to the health literacy challenge were allocated.	1) With the prior information a draft chronology of the release of national policy or position statements was developed.<br><br>2) Corres-ponding relevant health literacy practice change listed to chronology graph.	1) Themes describing OHL challenges & response, and chronology of events presented to Key Informants.<br><br>2) Feedback from Key Informants used to update the chronology of policies / position statements / reports and relevant practice change or response.<br><br>3) Identification of additional material which was added to the analysis and chronology.	1) Determine the processes taking place within the organisation because of the chronology (incremental systematic steps driven by national policy and group reflexivity).<br><br>2) Received last of the 2021 health information fact sheets.<br><br>3) PEMAT analysis commenced.	Analysis of themes from the material captured to determine the literacy challenges and OHL response finalised and sub-groups reduced to 4 major themes:<br><br>1. Audience Reach<br><br>2. Consistency and Branding<br><br>3. Person-Centred Language<br><br>4. Under-Standability and Actionability.	OHL challenges and OHL response themes tested with Key Informants and finalised.	PEMAT analysis finalised 17/6/2022.<br><br>Final approval of timelines and chronology of policies and position statements and relevant OHL practice change.	Draft manuscript of findings prepared.	Finalise article for publication.

Step 1	Conceptualisation and Development phase
Step 2	Literature and information search
Step 3	Monthly to bi-monthly key informants interviews
Step 4	Preliminary analysis
Step 5	PEMAT evaluation
Step 6	Finalised analysis of health literacy challenge and OHL response / actions
Step 7	Write up and dissemination
↑	Core Research team meetings (DM, GM, SL)

Table S2: Definitions of themes identified explaining health literacy challenge addressed by OHL response.

OHL Theme	Definition
**Audience reach**	To expand the reach to the target audience by reaching more priority groups through culturally and linguistically appropriate, age suitable, visually appealing and interactive health information material.
**Consistency and branding**	To align diabetes messaging about self-care across all health information materials and resources to promote reliability and as a mark of dependability.
**Person-centred language**	To incorporate language that puts the consumer first before their condition, while valuing the attitudes and experiences of people living with diabetes and respects the uniqueness of each individual.
**Understandability and actionability**	When health information material are understood by consumers of diverse backgrounds and varying levels of health literacy because they can process and explain key messages and actionable because they can identify what they can do based on the information presented.
**Group reflexivity**	Overt reflections of objectives, strategies and processes during group discussions as a mechanism for decision-making, focusing on widening the array of opinions considered to form a clear balance of advocacy and inquiry rather than decision-making harmony.

Figure S1: Health literacy challenges identified and OHL response.

Audience Reach

Narrative

In 2009, Diabetes Australia created a **strategy to diversify and produce more co-designed, culturally appropriate health information** to reach Aboriginal and Torres Strait Islander people and more people from culturally and linguistically diverse backgrounds (CALD).

Medical, Education and Scientific Advisory Council (MESAC) supported Diabetes Australia's ongoing work by promoting **ways to reduce health literacy demands** and to address the needs of a larger proportion of the Australian population with diabetes, including people from CALD backgrounds, in Feb 2013 by better articulating and understanding the target audience. MESAC appraisals highlighted areas requiring a change to meet health literacy. MESAC supported Diabetes Australia's **continuous quality improvement** by monitoring and identifying ways **to incorporate health literacy principles** and relevant stakeholder consultation.

NDSS Priority Area Leaders 'topics' became the 2013 Election platform and eventually a new national strategy.

National Diabetes Strategy Advisory Group in 2014 identified Goal 4, to reduce the impact of diabetes among Aboriginal and Torres Strait Islander peoples by developing and implementing culturally relevant programs in collaboration with their communities to improve health literacy and education and awareness about diabetes. Also, Goal 5 was to reduce the impact of diabetes among high-risk and vulnerable groups, including developing CALD appropriate diabetes education packages.

The **Australian National Diabetes Strategy 2016-2020** released in late 2015 promoted targeting CALD populations to increase access to care.

Department of Health and Diabetes Australia embarked on **the NDSS Enhancement Project** in 2018 using agile scrum methodology to improve access to NDSS resources, programs, services and products by improving **site navigation** for people with diabetes and their carers.

Organisational Health Literacy Response

- **NDSS Priority Area Leaders** were appointed in 2012 for older, younger, CALD, Aboriginal and Torres Strait Islander peoples, and psychosocial issues and diabetes in pregnancy. The 'Leaders', key senior health managers, advised the **NDSS Agents Group** (Diabetes Australia and state and territory organisation CEOs and NDSS General Manager) regarding priority groups. NDSS funding supported each NDSS Priority Leader related project/resource.
- **National Diabetes Strategy Advisory Group** appointed.
- NDSS **MESAC Administration Form** was created to facilitate a systematic process in April 2013. The form included: the title, action required (yes/no), deadline, target audience, background and purpose/reason for the development.
- An annual **NDSS Registrant Survey** from 2012, including questions about consumer fact sheets, usage and satisfaction.
- **Translation Projects**
  2009: First translated resources GDM DVDs in Turkish, Vietnamese, Simplified/Traditional Chinese and Arabic.
  2011: *10 for 10 Translation Project* into most common languages: Arabic, simplified Chinese, traditional Chinese, Greek, Italian, Korean, Spanish, Turkish, Urdu and Vietnamese.
  2016: *Translation Project* translated to 6 more languages.
  2018: Further translation of 4 more languages.
- Diabetes Australia appointed an **Aboriginal and Torres Strait Islander Engagement Manager** in 2018
- **NDSS Enhancement Project** delivered on phase 1 (NDSS Central), phase 2 (NDSS Health Professional Portal) and phase 3 (NDSS Registrants and Access Point Portal)

Consistency and Branding

Narrative

MESAC highlighted inconsistencies, font styles and colours during NDSS health information (programs/documents) reviews. The feedback was analysed and appraised for **trends** by Diabetes Australia as the NDSS Administrator.

MESAC and editor feedback was **critically discussed** quarterly, including objectives, strategies and processes between MESAC and Diabetes Australia that enabled **a reflective process. Themes** from feedback were used to develop and update the branding guide/standards and processes.

Organisational Health Literacy Response

- NDSS Brand developed in 2006 and **NDSS Brand Guide** in 2008
- The first **NDSS Style Guide** for authors was released in March 2015, following a significant overhaul of the branding document to include **health literacy and readability** assessment concepts with subsequent expansion and updates in 2016, 2017, 2018 and 2020.
- An updated **NDSS Brand Standards** was released in 2021.
- **National Consistency Policy** and National Consistency Taskforce in 2021 to reinforce the implementation of above guidelines.

Person-centred Language

Narrative

In 2009, Diabetes Australia's MESC promoted the need to meet the audience health literacy needs, noting that the fact sheets were written at the tertiary level.

Quarterly, from October 2013, MESAC feedback to reviewers from NDSS submitted products were **critically discussed** and analysed for **themes and trends** at meetings. Common themes from feedback and **open discussions about objectives, strategies and processes** informed NDSS documents. MESAC continually promoted the Diabetes Australia Position Statement about Language in feedback to authors.

Diabetes Australia led the 2021 **National Consistency Taskforce** about nationally consistent access to diabetes resources, programs and services.

Organisational Health Literacy Response

- Diabetes Australia released the **Position Statement: A New Language for Diabetes** - Improving communications with and about people with diabetes in July 2011, funded through the NDSS. The position statement was updated in May 2016.
- Diabetes Australia launched an updated **Position Statement: Our Language Matters** in 2021.
- **NDSS National Consistency Policy** requires that all new resources, programs and services involve co-design processes with target audiences, including end-user testing of all NDSS resources at the development level as an outcome of the National Consistency Taskforce.

Understandability and Actionability

Narrative

The **NDSS National Programs Group** (Diabetes Australia's NDSS team and state and territory organisations' health professional group) requested that **all resources be reviewed for health literacy** in 2012.

MESAC and NDSS Agents met in 2014 to agree on changes to the administration form used to inform MESAC of planned reviews. MESAC requested that **health literacy be addressed before DSME information** was forwarded for **appraisal and to promote awareness of health literacy needs to authors**. MESAC encouraged that materials were not accepted until authors addressed each area.

Throughout the 2016-21 NDSS Agreement, the MESAC continued to support Diabetes Australia to **embed a systematic review process; focusing on the readability and actionability of information** to ensure DSME information enacted an action. The NDSS now had a well-developed systematic quality improvement process where those submitting health information were exposed to and required to meet essential elements to ensure DSME information supported health literacy before submission. The MESAC appraisal process was critically reviewed regularly. These activities aimed **to promote national consistency, reduce duplication and improve health literacy**.

Organisational Health Literacy Response

- **Rapid Literature Review for Health Literacy** in people with diabetes was commission by ADEA (NDSS-funded) in 2014.
- **Improving Health Literacy for People with Diabetes practice recommendations** launched by ADEA (NDSS-funded) in March 2015
- **NDSS Administration Form**, which listed elements to promote health literacy, including a clear target audience, title styling, active voice use, readability scores (i.e. Flesch Kincaid to determine, e.g. reading ease and grade scores, average words per sentence and syllables) to meet the intended audience's needs. The form became a tool to guide authors in developing health literate diabetes information. The form requested evidence of the review process, the author/s' and expert advisory groups expertise to promote scientific and educational soundness.
- **Suite of digital DSME health information and programs**, delivered through a variety of channels and platforms, e.g., YouTube, podcasts, videos, animations, interactive presentations and online DSME programs through digital platforms and social media channels.
- **Diabetes-technology online educational materials and interactive resources** for people with diabetes and health professionals.
- **NDSS Preferred Language Checklist** launched in April 2021.

Table S3: List of English and translated diabetes fact sheets.

* First ten languages from 2010/11 Strategic Development Plan	Arabic*	Bengali	Chinese-S*	Chinese-T*	Dari	English	Farsi	Greek*	Hindi	Italian*	Karen	Khmer	Korean*	Macedonian	Nepali	Punjabi	Samoan	Sinha-lese	Somali	Spanish*	Tagalog	Tamil	Thai	Tongan	Turkish*	Urdu*	Vietnamese*
Understanding type 1 diabetes	X		X	X		X		X		X			X							X					X	X	X
Understanding type 2 diabetes	X		X	X	X	X	X	X		X	X		X	X	X	X	X		X	X		X	X	X	X	X	X
Understanding prediabetes						X																					
Managing worry about COVID-19 & diabetes	X	X	X	X	X	X	X	X	X	X	X	X	X	X	X	X	X	X	X	X	X	X	X	X	X	X	X
Diabetes care and COVID-19						X																					
Understanding gestational diabetes	X	X	X	X		X		X	X	X			X				X			X					X	X	X
Understanding gestational diabetes (video)		X				X	X					X				X										X	
Gestational diabetes: caring for yourself & your baby	X		X	X		X			X							X									X		X
Pregnancy and diabetes	X	X	X	X		X	X	X	X	X			X			X		X		X	X	X			X	X	X
Blood glucose monitoring	X		X	X	X	X	X	X		X	X		X				X			X		X			X		X
Continuous glucose monitoring						X																					
Flash glucose monitoring						X																					
Managing hypoglycaemia	X		X	X	X	X	X	X		X	X		X	X	X	X	X		X	X		X	X	X	X	X	X
Diabetes-related complications	X		X	X		X		X		X			X							X					X	X	X
Healthy meal ideas	X		X	X	X	X	X	X		X	X		X	X	X	X	X		X	X		X	X	X	X		X
Healthy snacks ideas	X		X	X	X	X	X	X		X	X		X	X	X	X	X		X	X		X	X	X	X		X
Eating out and diabetes						X																					
Making healthy food choices	X		X	X	X	X	X	X		X	X		X	X	X	X	X		X	X		X	X	X	X	X	X
Healthy cooking ideas						X																					
Carbohydrate counting						X																					
The glycaemic index						X																					
Alternative sweeteners						X																					
Alcohol and diabetes						X																					
Understanding food labels						X																					
Physical activity	X		X	X	X	X	X	X		X	X		X	X	X	X	X		X	X		X	X	X	X	X	X
Your diabetes annual cycle of care	X		X	X		X		X		X	X		X	X	X	X	X		X	X			X	X	X		X
Looking after your feet						X																					
Looking after your dental health						X																					
Looking after your eyes						X																					
Looking after your kidneys						X																					
Looking after your heart						X																					
Sexual health and diabetes						X																					
Diabetes and driving: a quick guide	X		X	X	X	X	X	X		X	X		X	X	X	X	X		X	X		X	X	X	X		X
Diabetes and driving: a quick guide video	X		X	X	X	X	X	X		X	X		X	X	X	X	X		X	X		X	X	X	X		X
Type 2 diabetes: a quick guide	X		X	X	X		X	X		X	X		X	X	X	X	X		X	X		X	X	X	X		X
Medications for type 2 diabetes						X																					
Insulin and diabetes						X																					
Concerns about starting insulin (type 2 diabetes)						X																					
Adjusting to life with diabetes						X																					
Diabetes and anxiety						X																					
Diabetes distress						X																					
Diabetes and depression						X																					
Diabetes and disordered eating						X																					
Fear of hypoglycaemia						X																					
When and how a psychologist can support me	X		X	X	X	X	X	X		X	X		X	X	X	X	X		X	X		X	X	X	X		X
Living well with type 1 diabetes – what to do when you are sick						X																					
Living well with type 2 diabetes – what to do when you are sick						X																					
Surgery and hospital stays						X																					
Polycystic ovarian syndrome						X																					
Coeliac disease and diabetes						X																					
Steroid medication and diabetes						X																					
Travel and diabetes						X																					
My diabetes plan for natural disasters & emergencies	X		X	X		X																			X		X
Live well & save money with the NDSS card	X		X	X																					X		X
Live well & save money with the NDSS card (poster)	X		X	X																					X		X
Peer support for diabetes						X																					
Caring for someone with diabetes (family & friends)						X																					
Annual cycle of care podcast series					X		X															X					
TOTAL	22	4	21	21	13	54	15	17	4	17	13	2	17	12	12	15	14	2	12	17	2	14	12	12	21	10	21

Figure S2: Medical Education and Scientific Advisory Council Requests for Resource Review.

NDSS National Diabetes Services Scheme — An Australian Government Initiative

NDSS Helpline 1800 637 700
ndss.com.au

diabetes australia — The NDSS is administered by Diabetes Australia

Medical Education and Scientific Advisory Council Requests for Resource Review

**Resource Title:**<br>*e.g. Fact Sheet: Blood glucose monitoring*		
**Reason for Review**		
☐ New Resource	☒ Existing Resource	Reason for development/update:<br>• *e.g. scheduled for regular biannual review to ensure content is aligned to current evidence and recommendation*<br>• *e.g. revision of language and verbiage for alignment with Diabetes Australia position on language, NDSS preferred language statement and the NDSS Style Guidelines*
☐ Initial Review	☒ Subsequent Review	Issues amended in subsequent review:

**Resource Type**				
☐ Booklet	☐ Brochure	☐ Communications	☒ Fact sheet	☐ Handbook
☐ Module	☐ Podcast	☐ Program material	☐ Web content	☐ Video
☐ Other:				

**Resource Description**
e.g. The NDSS Fact Sheets are a suite of resources aimed at people living with diabetes, their carers and the community at large. Fact Sheets are core NDSS resources that have been through multiple iterations and reviews since their inception. They have a high brand recognition and is frequently used as an adjunct resource by health professionals during consultations with people with diabetes.<br>*Fact Sheets are used by NDSS Agents during service delivery and provided to health professionals on request with a reported average of 15,000 supplied every month. In addition to this, approximately 21,000 Fact Sheets are accessed, a mixture of download and views from the NDSS website every month.*<br>*The suite of Fact Sheets is scheduled for a biannual review.*

Version 3 July 2020.

Related Resources

☐ NDSS:

☐ Non NDSS:

Target Audience

Consumers: *(Select all that apply)*	☐ Type 2 Diabetes	☐ Registrant	☐ Carer	☐ Child	☐ Adolescent
	☐ Type 1 diabetes	☐ Elderly	☐ Adult	☐ Public	☐ Pregnancy
	☐ Gestational diabetes	☐ Other:			
Health Professional: *(Select all that apply and practice sector where applicable)*	☐ General Practitioner	☐ Optometrist	☐ Nurse	☐ Dietitian	☐ Physiotherapist
	☐ Diabetes Specialist	☐ Podiatrist	☐ Dentist	☐ Pharmacist	☐ Paediatrician
	☐ Diabetes Educator	☐ Other:			

Language Review

☒ Flesch-Kincaid Score; *See below table*	☐ Professional Editorial Review	☒ Diabetes Australia Language Position	☒ NDSS Preferred Language	☒ NDSS Style Guide

☒ Other:

**Name of document**	**F-K Reading Ease Score**	**F-K Reading Grade Score**	**Reading level**	**Av. Words per sentence**	**Ave syllables per word**
Mock Example	66.3	7.3	8-9 Grade (Plain English)	13.4	1.5
Mock Example	67.1	7	8-9 Grade (Plain English)	12.6	1.5
Mock Example	59.2	8	10-12 Grade (Fairly difficult to read)	12.1	1.6
Mock Example	59.4	7.9	10-12 Grade (Fairly difficult to read)	11.9	1.6

Further Comments:

e.g. while the Flesch - Kincaid score has been improved, some fact sheets may have a higher rating due to the technical and medical terminology that is necessary to incorporate.

Resource Developer	
Author (s):<br>*(Please provide the details of all contributors to the development. Attach details as required.)*	Name: *Original author unknown. Input of various health professionals over time*
	Qualification:
	Professional Practice Area:
	Area(s) of expertise:
	Name:
	Qualification:
	Professional Practice Area:
	Area(s) of expertise:
	Element/section developed:
	Name:
	Qualification:
	Professional Practice Area:
	Area(s) of expertise:
	Element/section developed:
Initial Reviewer(s):<br>*(Please provide the details of all reviewers. Attach details as required.)*	Name:
	Qualification:
	Professional Practice Area:
	Area(s) of expertise:
	Review of:
Peer Reviewer(s):<br>*(Please provide the details of all reviewers. Attach details as required.)*	Name:
	Qualification:
	Professional Practice Area:
	Area(s) of expertise:
	Review of:

Contribution from Internal Stakeholders	
☐ Diabetes Australia:	
☐ National Advisors:	
☐ Agents:	
☐ ERG:	
☐ Working Group:	
☐ National Leads:	
☐ Other:	
**Contribution from External Stakeholders**	
☐ Consumer Review:	
☐ Health Professional Review:	
☐ Other:	
**References and Aligned Publications**	
**List of Accompanying Documents**	
**Form Completion**	
Name:	Position:
Date:	Signed:

Processing	
**Received:**	**Submitted:**
**Name:**	**Position:**
